# Considering the Microbiome in Stress-Related and Neurodevelopmental Trajectories to Schizophrenia

**DOI:** 10.3389/fpsyt.2020.00629

**Published:** 2020-07-03

**Authors:** Kevin W. Hoffman, Jakleen J. Lee, Cheryl M. Corcoran, David Kimhy, Thorsten M. Kranz, Dolores Malaspina

**Affiliations:** ^1^ Department of Psychiatry, Icahn School of Medicine at Mount Sinai, New York, NY, United States; ^2^ James J. Peters VA Medical Center, Mental Illness Research, Education and Clinical Centers (MIRECC), New York, NY, United States; ^3^ Department of Psychiatry, Psychosomatic Medicine and Psychotherapy, University Hospital, Goethe University, Frankfurt, Germany

**Keywords:** schizophrenia, microbiome, brain-derived neurotrophic factor, development, stress, cortisol

## Abstract

Early life adversity and prenatal stress are consistently associated with an increased risk for schizophrenia, although the exact pathogenic mechanisms linking the exposures with the disease remain elusive. Our previous view of the HPA stress axis as an elegant but simple negative feedback loop, orchestrating adaptation to stressors among the hypothalamus, pituitary, and adrenal glands, needs to be updated. Research in the last two decades shows that important bidirectional signaling between the HPA axis and intestinal mucosa modulates brain function and neurochemistry, including effects on glucocorticoid hormones and brain-derived neurotrophic factor (BDNF). The intestinal microbiome in earliest life, which is seeded by the vaginal microbiome during delivery, programs the development of the HPA axis in a critical developmental window, determining stress sensitivity and HPA function as well as immune system development. The crosstalk between the HPA and the Microbiome Gut Brain Axis (MGBA) is particularly high in the hippocampus, the most consistently disrupted neural region in persons with schizophrenia. Animal models suggest that the MGBA remains influential on behavior and physiology across developmental stages, including the perinatal window, early childhood, adolescence, and young adulthood. Understanding the role of the microbiome on critical risk related stressors may enhance or transform of understanding of the origins of schizophrenia and offer new approaches to increase resilience against stress effects for preventing and treating schizophrenia.

## Introduction

Schizophrenia presents an enormous burden to individuals, families, communities, and public health, but the mechanisms underlying its pathogenesis, presentation, and course remain largely enigmatic, with no interventions known to prevent or cure the disease. New perspectives are necessary to overcome this roadblock. The microbiome, which broadly refers to the collection of genomes of the commensal microbes inhabiting our bodies, influences our health in broad and complex ways. The emerging science of the microbiome is a promising new domain that could shed light on crucial disparate features of schizophrenia, including its association with prenatal and life course stressors, neurodevelopmental underpinnings, inflammatory neuropathology, particularly of the hippocampus and its metabolic comorbidity.

### The Microbiome

The microbiome comprises a dynamic ecological community of commensal microorganisms that inhabit our body where it interfaces with the environment. These specific microbes, which are collectively referred to as the microbiota, consist of bacteria, viruses, fungi, and protozoa; approximately equal our own cells in number; and combined pose over 200 times the number of genes as the human genome (1, reviewed in 2). Recent advances in high-throughput genetic sequencing and computational abilities reveal the richness, complexity, and essential role of the microbiome in human health. Its composition varies by anatomic region, with the gut microbiome in the distal large intestine considered the most influential for health.

After being seeded at birth by maternal vaginal bacteria in the birth canal, the neonate gut microbiota develops in a phasic manner, largely due to feeding. The gut is initially colonized by microaerophilic *Proteobacteria* and facultative anaerobic *Actinobacteria*, which consume oxygen and create a suitable niche for subsequent obligate anaerobes like *Bacteroides*, *Clostridium*, and *Bifidobacterium* spp. (3, 4) Breast milk stimulates the growth of bifidabacteria, but weaning results in the emergence of *Firmicutes* and *Bacteroidetes* (5). These phyla proliferate with the introduction of solid foods and eventually come to dominate the gut microbiota (5). By 2.5 to 3 years of age, the infant gut microbiota structure stabilizes and resembles the adult gut microbiota, which is also dominated by *Firmicutes* and *Bacteroidetes* (3). The developmental dynamics of the infant gut microbiota are shaped by host genes, host immunity and environmental factors, such as diet, medications, and climate (6–8).

Over the last decade, it has emerged that the human microbiome highly influences the development of the central nervous system (CNS) and the immune system. The microbiome is shaped by stress exposures from early life and, in turn, influences stress responsivity (9). Given this new information, our models of the endocrine modulation of the stress response should be updated to account for the microbiome.

**Figure 1:**
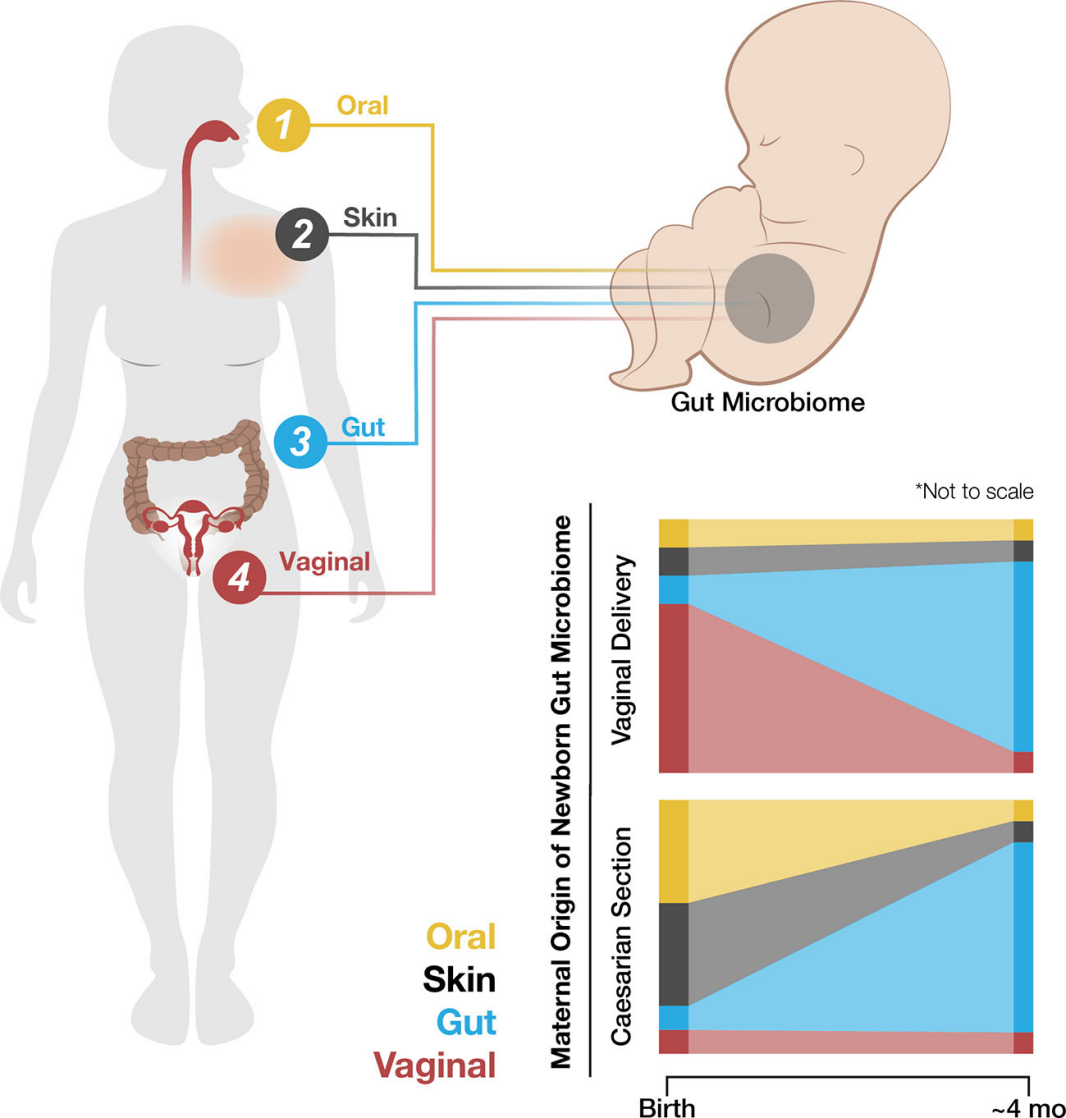
The hypothalamic-pituitary-adrenal (HPA) axis regulates the response to stress (red lines). Stress activates the hypothalamus to secrete cortisol-releasing hormone (CRH), which induces the anterior pituitary gland to release adenocorticotropin hormone (ACTH; solid red lines). ACTH stimulates the adrenal gland cortex to produce cortisol (solid red line), which negatively regulates CRH production to terminate the stress response cascade (dashed red line). Excess or chronic stress can disturb normal HPA axis function *via* altered neuroendocrine signaling and gut dysbiosis (blue). Under excess or chronic stress, the hypothalamus is hyperactivated, leading to upregulation of the anterior pituitary gland and adrenal gland activation (plus signs) as well as downregulation of CRH inhibition (minus sign). Consequently, abnormally high levels of cortisol result in increased hippocampal signaling, which may overactivate the hippocampus, cause inflammation, and alter the crosstalk equilibrium between cortisol and BDNF in the hippocampus. Excess or chronic stress causes gut dysbiosis, which alters gut hormone and microbe metabolite signaling from the gut to the brain through the vagus nerve, i.e., the GBA.

The bidirectional influence of the gut microbiome and CNS occurs through the “gut-brain axis” (GBA), components of which include the vagal nerve, gut hormone signaling, immune system, tryptophan metabolism, and microbial metabolites, such as short-chain fatty acids (reviewed in 10). Activity along the GBA intersects with the HPA axis (**Figure 1**) and may influence many psychiatric disorders, as evidenced by the association of gut dysbiosis with autism, depression, and anxiety disorders as well as functional gastrointestinal disorders (11–16). Given the purported inflammatory underpinnings for schizophrenia and its severe comorbidities with other microbiome-linked metabolic diseases, associations between schizophrenia and the microbiome are of great interest.

#### Stress Response and the Microbiome

Awareness of the overlap of stress signaling and the microbiome began in 2004 with the report that germ-free mice had an exaggerated hypothalamus-pituitary-adrenal (HPA) responses to stress in comparison to non-germ-free mice (17). The “microbiome-gut-brain axis” (MGBA) refers to bidirectional signaling between the gut flora and CNS. Acute and chronic stressors that activate the HPA axis also influence the microbiome and gut epithelium which participate in behavioral and systemic stress effects. The gut and brain communicate through the vagal (parasympathetic) nerve, which is a cholinergic anti-inflammatory pathway associated with slowed heart and respiratory rates and digestive function. Under stress, the sympathetic nervous system predominates and vagal function is reduced. The gut microbiome produces neurotransmitters that influence behavior, including acetylcholine, catecholamines, γ-aminobutyric acid, histamine, melatonin, and serotonin, all of which are also essential for regulating gastrointestinal peristalsis and sensation. Thus, the HPA axis and GBA are intersecting, co-dependent loops for managing stress and inflammation as part of their physiological function.

In this review, we illuminate aspects of the stress response and the microbiome as the GBA, with respect to schizophrenia. The impact of stress exposures on the brain will almost certainly entail signaling with the microbiome. Some factors that are associated with an increased risk for schizophrenia are considered across developmental stages, including the perinatal window, early childhood, adolescence, and young adulthood.

### The HPA Axis, Hippocampus, Neurotrophins, and Schizophrenia

The neurobiology of the stress cascade and its potential for toxicity is well described. The HPA axis is the stress response system through which stress hormones and the CNS interact. Early dysregulation of the HPA axis is associated with adult stress-related disorders, including schizophrenia (18–20). Mechanistically, HPA axis dysregulation is considered to be linked to schizophrenia risk *via* glucocorticoid (GC) overproduction, especially during vulnerable phases of neurodevelopment. Cortisol-releasing hormone (CRH) is released from the paraventricular nucleus of the hypothalamus following physical or psychological stressors. CRH binds receptors on the anterior pituitary gland, driving release of adrenocorticotropic hormone (ACTH). This stimulates the adrenal cortex to release cortisol, the human GC hormone. Under physiological conditions increasing cortisol levels inhibit CRH release, terminating this stress cascade through a negative feedback loop. However, excess and chronic stress hyperactivate the HPA axis and cause abnormally high GC levels (21–24).

The effect of elevated GC levels on the hippocampus, the essential structure for memory and contextualizing new information, may be relevant. The hippocampus is the most commonly abnormal brain region in groups of schizophrenia cases, with progressive hippocampal volume loss a common observation (25). Increased activation, metabolism, and inflammation of the anterior hippocampus are associated with psychotic symptoms (26, 27) (reviewed in 28). The hippocampus possesses a high concentration of GC receptors that promote threat appraisal and help organize the stress response. Increased GC levels may drive overactivation and inflammation of the hippocampus and thereby promote schizophrenia (reviewed in 29–31).

GCs may also influence schizophrenia through interaction with neurotrophin pathways. Neurotrophins are growth factors responsible for neuron growth, differentiation, and formation of new synapses (32). Brain-derived neurotrophic factor (BDNF), the most abundant neurotrophin, is highly active in the hippocampus, cortex, and basal forebrain, where it binds its receptor, tyrosine kinase receptor B (TrkB), to play a key role in synaptic plasticity and long-term memory formation (33). Because GC receptors and TrkB are co-expressed in the hippocampus, important crosstalk between GCs and BDNF occurs here, as threat appraisal relies on both current stress and appropriate context from memory (34). As such, GC and BDNF equilibrium remains crucial for stress response regulation throughout life. Impairment of GC receptors and TrkB in the hippocampus favors vulnerability to stress-related disorders, including schizophrenia (reviewed in 35).

These pathways are influenced by the microbiome. Gut dysbiosis can indirectly influence cortisol release and sensitivity *via* chronic cytokine-mediated inflammation (36–38). This proinflammatory state may be driven by microbes crossing the intestinal barrier, releasing microbial byproducts such as lipopolysaccharide (LPS), or be moderated through bacterial metabolites, such as short-chain fatty acids (39–43) (reviewed in 44). The microbiome further influences the structure and function of the amygdala, which is critical for emotion learning and social behavior, especially responses linked to anxiety and/or fear (45, 46). Studies of germ-free mice show that the absence of the microbiome during early critical developmental windows leads to chronic cortisol elevation and altered hippocampal BDNF levels (17, 47). Depleting the microbiome of previously healthy mice through antibiotics disrupts the HPA axis (36, 48, 49). Taken together, these findings suggest that a healthy microbiome is an important component of HPA axis development and that early alterations of the microbiome can affect neuroendocrine pathways throughout life.

### Resilience in Schizophrenia

Identifying factors to increase resilience against stress is an area of active research that may be addressed through MGBA research. Anxiety and depression-like symptoms in germ free animas as well as the transference of a depression phenotype from a human patient to a rats through fecal microbiota support the feasibility of this approach (50). Mice deficient in the CRH_1_ receptor and those with increased GR activity display more resilient behaviors (51–54) and these hormones can be modulating by the gut microbiome (11, 50). Likewise, the expression of serotonergic, glutamatergic, and GABA, which are dysregulated in association with poor resilience (55), are modulated through microbiome effects in animal models (reviewed in 56). A healthy microbiome may also contribute to resilience through emotion regulation that manifests as positive emotions and optimism, cognitive flexibility, and healthy interpersonal function, attributes that are associated with active coping styles (reviewed in 57). There may be treatment role for nutritional supplementation, as stress-related behaviors and HPA dysfunction in socially isolated male mice was remedied by dietary supplementation with DHA (58) and a rat study even demonstrated that stress sensitivity from early life trauma might be remediated through long-term supplementation with an eicosapentaenoic acid (EPA)/DHA mixture (59). The overlap of findings on the M-GBA with neuroendocrine and behavioral measures with those implicated for resilience indicate opportunities to modify the impact of stress exposures and augment resilience by targeting the microbiome.

## Perinatal Development

### Introduction

In 1934, Rosanoff and colleagues published “The Etiology of So-Called Schizophrenic Psychosis” in the American Journal of Psychology (60). This manuscript, which examined 142 pairs of twins either concordant or discordant for schizophrenia, was the first to associate birth complications with schizophrenia. In subsequent decades, schizophrenia risk during pregnancy, birth, and the neonatal period was broadly examined. Many risk factors were identified that occurred in important early developmental stages, including maternal infection, stress, and medical complications during pregnancy, fetal growth restrictions, and hypoxia during pregnancy and birth. Overall, early-life exposures have the greatest impact on the development and function of central neural circuits and the immune system (46).

Missing from this well-developed story is the impact of maternal exposures on her microbiome and the potential for vaginal dysbiosis (61, 62). The newborn’s gut microbiome is seeded by the maternal vaginal microbiome during passage through the vaginal canal (8, 63). Disruptions in maternal microbiome may cause the newborn to be seeded with a more inflammatory gut microbiome (64, 65). It is this newborn microbiome that appears to have a strong influence in driving the development of the immune system and directing neurodevelopment (17, 66–70). These important contributions to fetal development must now be included is considering the action of schizophrenia risk factors in the perinatal period.

### Maternal Infection

Maternal infection during pregnancy is associated with the risk for schizophrenia and is a maternal stressor. A 1988 study reported an increased rate for persons who were *in utero* during the 1957 influenza epidemic (71). Subsequent studies replicated this finding and suggested the second trimester as the gestational risk period for schizophrenia from influenza infection, although other evidence points to the first trimester (72–74). Other maternal infections associated with the offspring’s risk for schizophrenia include rubella, varicella zoster virus, herpes simplex virus, and *Toxoplasma gondii*, known as TORCH agents, which can cross the placental barrier and directly infect the fetus, as can measles, polio, bacterial bronchopneumonias, and infections of the genitals and reproductive tract (75–77). Taken as a whole, infection with this group of pathogens during pregnancy is relatively common and may be an important factor for psychiatric disorder risk.

As to mechanism, there are several possibilities. One of these is direct invasion, which is consistent with the very high rate of schizophrenia following prenatal rubella, up to 20%, given rubella’s well-known propensity for neural invasion in the developing fetus (76). Supporting invasion, a mouse model of influenza infection showed persistence of influenza RNA in the brains of offspring of infected pregnant mice (78). Another possibility is indirect damage driven by maternal inflammation. During maternal infection, inflammatory cytokine levels are elevated (75) and these may disrupt fetal neurodevelopment and potentially drive schizophrenia risk. For instance, the proinflammatory cytokine IL-1β negatively regulates hippocampal neurogenesis, suggesting a possible mechanism through which chronic inflammation could affect schizophrenia susceptibility (79). Notably, maternal inflammation correlates with later childhood psychiatric symptoms (80). Other potential risk pathways include effects from maternal fever, maternal antibodies crossing the placenta and medications, such as analgesics and anti-inflammatories, taken by the mother during infection, all of which may impact fetal neurodevelopment (81–83).

However, maternal infections also alter her microbiome, potentially leading to increased production of inflammatory products released by her gut, as well as to disrupted seeding of the neonatal microbiome at birth (64, 65). Neonates born to mothers with ongoing HIV infection show decreased gut microbiome diversity including reduced levels of *Prevotella*, a bacterial genus linked to inflammatory regulation of stressor (84). It is possible that dysbiosis secondary to maternal infection sensitizes the neonate to further stress-related injury, including elevated schizophrenia risk. Given the data demonstrating the impact of maternal inflammation on offspring schizophrenia risk and the microbiome’s potential contributions to this inflammation, the microbiome may be a key player in schizophrenia pathogenesis.

### Maternal Stress

Maternal stressors, such as depression, unwanted pregnancy, death of a partner, and exposure to war and disasters, are associated with schizophrenia in offspring (19, 85–88). For female fetuses, these external stressors are most strongly correlated with schizophrenia when they occur during the first trimester; however, male fetuses demonstrated increased schizophrenia risk through the second trimester, suggesting sex differences in critical periods (87, 88). Importantly, maternal stress during the first six months of postnatal life is associated with worse behavioral outcomes in children, suggesting that disrupted caregiving may also be a component to the schizophrenia risk posed by maternal stress (89). Additionally, prenatal nutritional deficiencies, including gross calorie deficits during famine and micronutrient deficiencies in homocysteine and vitamin D, are associated with both schizophrenia and the above-mentioned stressors (90–94), which certainly impact the microbiome composition. The short-term effects of maternal stress may act through adverse pregnancy outcomes, while the long-term effects on neurodevelopment may involve altered neonatal stress programming and gut dysbiosis (95). Maternal stress increases fetal and neonatal exposure to maternal cortisol, altering growth and behavior in humans and animal models (reviewed in 96). Stress also has well-documented effects on the microbiome, which may in turn alter inflammation and neurodevelopment in a developing neonate (62, 97–103). As an example, maternal perinatal stress increases offspring susceptibility to allergic diseases, which suggests interactivity between maternal GCs, perinatal immune development, and possible maternal dysbiosis (79). In a mouse model, prenatal maternal stress led to dysbiosis in both mother and offspring, increased IL-1β *in utero*, and a corresponding decrease in BDNF in offspring (104). Other experiments have shown antibiotics alter BDNF levels in dysbiotic mice, suggesting that interventions in the gut microbiome may be important in modifying risk (105).

Exploring how the maternal stress influences her microbiome for fetal effects relevant to schizophrenia risk may enhance our understanding of the disease and suggest new treatments or prophylactics through probiotic use (reviewed in 106). Mechanistically, the microbiome-driven effects of stress may manifest through alterations of the HPA axis during key developmental stages (107), impaired development of small intestine immune tissue and IgA production (108, 109), or alterations in gut-metabolites leading to aberrant development (110). Given that many of these downstream events are linked with schizophrenia risk, future work should aim to elicit the microbiome contributions of schizophrenia risk secondary to maternal stress.

### Fetal Hypoxia

Many obstetric complications can lead to fetal hypoxia, which carries well-known risks to medial temporal regions. With regards to schizophrenia, fetal hypoxia may be the most significant risk factor among obstetric complications, in addition to maternal infections and fetal growth restriction (111, 112). Multiple studies report increased exposure to fetal hypoxia among persons with schizophrenia (113–115). One study show fetal hypoxia predicts the risk for early onset schizophrenia even after controlling for prenatal infection and fetal growth restriction (116). Further, fetal hypoxia is associated with reduced gray matter and ventricular enlargement in cases with schizophrenia and their non-ill siblings, although not in unrelated controls (117). Mechanistically, hypoxia may have an additive effect with genetic factors hastening the onset of schizophrenia in susceptible individuals (118). Certainly hypoxia may influence the composition and function of the gut microbiome (119, 120). As described above with infection and stress, these alterations increase future susceptibility to stress by influencing systemic inflammation, stress pathways, and BDNF production. Additionally, maternal microbes may invade the fetal brain following a hypoxic episode, as has been shown in sheep (121).

### Fetal Growth Restriction

In 1966, a small but significant reduction in birth weight was observed in schizophrenic patients when compared to their siblings (122), prompting consideration that fetal growth restriction was a schizophrenia risk factor. Some, but not all studies associated lower birth weight, reduced head circumference, and congenital malformations with increased schizophrenia risk (123). There are heterogeneous causes of fetal growth restriction, only some of which may be associated with the risk for schizophrenia (124).

### Maternal Complications

Other perinatal obstetric complications include maternal bleeding, maternal diabetes, preeclampsia, and caesarean section birth complications (125–127) (reviewed in 128). These perinatal traumas—along with the aforementioned factors of maternal infection, maternal stress, fetal hypoxia, and fetal growth restriction—altogether present a compelling argument for a close connection between the early window of neural development and schizophrenia risk. Recent advances indicate that the vaginal microbiome suggest that it may be a key player in this relationship. After all, these traumas occur during the perinatal period, when initial microbiota seeding of the newborn’s gut by the maternal vaginal microbiome occurs during fetal passage through the birth canal.

### Neuroendocrine Pathways

Cortisol, the primary human “stress” hormone, is also of central relevance for the developing fetus, promoting the maturation of vital organs, including the lungs, gastrointestinal tract, liver, heart, and brain. As such, the fetal HPA axis is tightly regulated, and is responsive to minute changes in fetal plasma levels of cortisol, which easily crosses the placental barrier (reviewed in 129). Due to their high cortisol sensitivity, developing fetuses rely on the placental enzyme 11β-hydroxysteroid dehydrogenase type 2 (11β-HSD2) to inactivate maternal cortisol by converting it to less active cortisone, beginning in the second trimester (130). Thus, in early gestation, before placental 11β-HSD2 is induced, maternal hypercortisolemia has potent effects on developmental gene expression. Even after the induction of 11β-HST2, some cortisone can be reactivated through 11β-hydroxysteroid dehydrogenase type 2 (11β-HSD2), which converts cortisone back to cortisol (reviewed in 131). This effect can be heightened by factors like maternal protein malnutrition, which diminish 11β-HSD2 gene expression (132). The detrimental effects of elevated exposure to maternal cortisol go beyond fetal development to influence emotional and behavioral disturbances during infancy and childhood and in later life (85) possibly including the perinatal schizophrenia risk pathways described above (reviewed in 133). Beyond the direct association between maternal stress and schizophrenia, elevated maternal cortisol may enhance other risks. In one study, elevated maternal cortisol during the second trimester enhanced the risk for adolescent onset depression in the offspring of mothers who experienced infections during pregnancy (134).

The neurotrophin BDNF is also critical for neurodevelopment. Elevated levels of BDNF are reported in fetuses with severe growth restriction as well as those with macrosomia in the context of maternal diabetes (135). Mechanistically, it is proposed that BDNF is neuroprotective in the developing fetus through anti-inflammatory mechanisms (136). *In vivo* animal models demonstrate that BDNF can reduce hypoxic brain injury through modulation of inflammatory cytokines and promotion of microglial activation (137). Given BDNF’s protective role in the developing brain, it is possible that downregulation of BDNF could exacerbate schizophrenia risk in the perinatal window.

### The Microbiome

**Figure 2:**
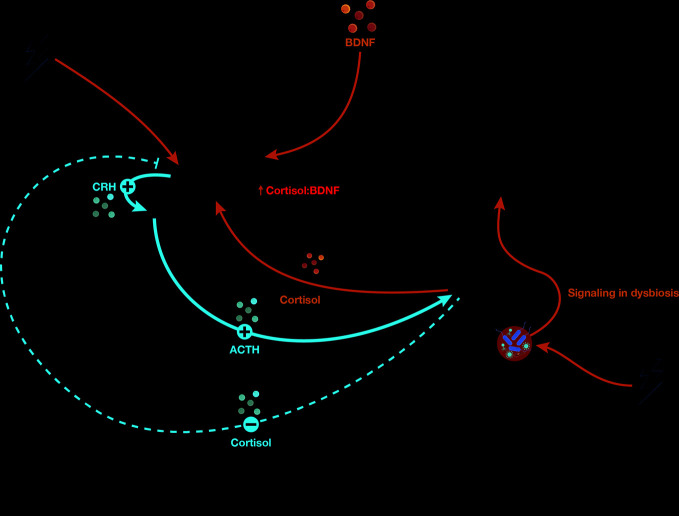
The newborn neonate gut initially contains bacterial strains from the mother’s oral, skin, gut, and vaginal microbiomes. The maternal source of initial colonization varies by the method of fetal delivery, i.e., vaginal birth or caesarean section. Although the newborn neonate gut microbiome stabilizes to resemble the mother’s gut microbiome by about four months of age, this early and transient variability may have long-term impacts on childhood development.

Colonization of a newborn neonate gut is normally seeded by the vaginal microbiome during birth, as described, along with maternal vaginal, skin, and oral and fecal bacterial strains (8, 63, 138). These vaginal contributions are transient and by four months post-birth, the infant’s gut microbiome is more similar to the maternal gut microbiome (**Figure 2**) (139, reviewed in 140). Neonates born *via* caesarean section lack exposure to the maternal vaginal microbiome and demonstrate a higher prevalence of maternal oral and skin microbes. They are also more likely to develop immune-related disorders (8, 64, 65, 141, 142). Disruptions of the maternal vaginal microbiome *via* infection, stress, or other pathways may lead to neonatal dysbiosis (65, reviewed in 143). Pre-term birth, caesarean sections, steroid use, and antibiotic use are also associated with dysbiosis in the newborn infant (144, 145).

The initial development of the microbiome, including its seeding at birth and development through very early childhood, is important for the development of a healthy core microbiome that is resistant to later perturbation. Given that dysregulation of the microbiome can cause pathogenic inflammation, dysbiosis in the perinatal window may lead to long-term inflammatory dysregulation (146–148). Further studies are needed to determine how maternal flora may influence immune development and schizophrenia risk in their offspring.

## Early Childhood

### Introduction

Childhood onset of schizophrenia is rare, but a number of neurologic and psychiatric features are already present in childhood. Likewise, a number of traumatic exposures in childhood are associated with schizophrenia risk. The possibility that these presentations reflect the interactions of stress signaling and neurotrophic effects that may be influenced or modulate by the microbiome effects, which is currently being explored.

#### Signs Across Neurodevelopment

Schizophrenia is highly heterogeneous and no one developmental trajectory can describe the risk pathway for all cases. However, the literature does note certain clusters of behavioral features, including gross motor dysfunction and lower verbal intelligence (149–153). As children develop, personality traits, social behaviors, and mood symptoms may emerge that are more imminently related to the risk for psychosis (reviewed in 154).

During the first two years of life, infants undergo rapid neurodevelopment achieving important motor milestones, like walking, running, pointing, stacking blocks; language milestones, like simple sentences and phrases from a vocabulary of hundreds of words; and social milestones, like beginning self-sufficiency, responding to requests, recognizing self in photographs (reviewed in 155). As failure to achieve milestones raises concern for neurologic development, this developmental stage has been studied in the offspring of affected mothers, in whom a 10% recurrence risk is anticipated. “Pandysmaturation’ was identified as a risk predictor in these “high risk” offspring, which involves a delay in cranial development and visual-motor development and disorganized motor performance (156, 157). Other studies identified passive infants with short attention spans, absence of stranger anxiety, poor communication competence, or abnormal use of language, and lower reactivity as signs of increased schizophrenia risk (158–162).

As children grow into early childhood, they typically become more coordinated, speak fluently, begin to learn reading and writing, and form friendships and social circles. Here again motor difficulties including clumsiness, poor coordination, and poor balance are predictive of higher schizophrenia risk (163, 164). Academically, learning disabilities like dyslexia are associated with higher schizophrenia risk (162). Among children of individuals with schizophrenia, relative decreases in coherence and complexity of language are associated with later schizophrenia risk (165). Socially, isolation, impaired affection, disturbed behavior, hyperactivity, impulsivity, and mood dysregulation including depressive signs and emotional lability are concerning for increased schizophrenia risk (158, 161, 162, 164).

In later childhood before transitioning into adolescence, children continue to improve in athletic, academic, and social behavior. Motor impairment of coordination and balance may become more striking in children with high schizophrenia risk (166–168). Additionally, high risk children may display learning difficulties in attention, concentration, memory, and thought as well as behavioral and mood dysfunctions such as increased aggression, problematic interpersonal relations, social isolation, low self-esteem, offending behaviors, poor affective control, and depression (152, 153, 162, 169–176).

Taken together, childhood impairments in neurologic development, marked by motor, cognitive, and behavioral disturbances, appear be on the trajectory toward schizophrenia, although most children with these features will not become psychotic. Notably, many of these factors entail stress effects on neuroendocrine function and neural plasticity. New research tracking the microbiome over development is showing its role in neurodevelopment and behavioral responses.

#### Exposure to Trauma

Traumatic experiences, ranging from abuse to accidental injuries, serious infections, and hospitalizations, may increase risk for schizophrenia (reviewed in 177). Trauma that occurs in childhood and adolescence is associated with psychosis and other psychiatric outcomes. Neurobiological studies demonstrate a stress hyporesponsive period in humans during the 6^th^ through 12^th^ postnatal months. Adverse experiences of newborns during this period can have lasting effects on HPA axis modulation (178, 179) from a long term elevation of basal GC secretion. Early life stress (ELS) rodent experiments demonstrate that maternal separation effects on stress sensitivity are mediated through GC-dependent mechanisms (reviewed in 180).

BDNF genetic variants may also influence sensitivity to trauma. There are many variants to the human BDNF gene, however, relatively few common variants fall within coding regions (181). Among these the BDNF^Val66Met^ variant is the most studied overall and has been specifically investigated with regards to schizophrenia risk (reviewed in 182). The BDNF^Val66Met^ polymorphism disrupts episodic memory in humans as a hippocampus-dependent memory function. Extensive studies in both animal models and humans have explored the effects of this polymorphism on numerous psychiatric disorders (reviewed in 183). Regarding schizophrenia risk, the 66Met allele decreases BDNF release probability (184), producing lower efficiency in neurotrophic activity, which is required for neurogenesis and neuroplasticity (185). It is associated with impaired episodic memory and lesser hippocampal activation (186). 66Met carriers with schizophrenia spectrum or bipolar disorders exposed to childhood sexual abuse show reduced grey matter volumes, consistent with the reduced BDNF mRNA levels in 66Met carriers who were exposed to childhood sexual abuse (186).

The higher sensitivity to trauma among 66Met carriers may be explained through the physiopathology of stress-induced changes in neural systems. BDNF plays a key role in neuronal plasticity (32, 187). BDNF-signaling is impaired by ELS; early traumas can evoke significant memory impairments in adulthood in association with reduced BDNF levels (188). This reduction, explained by hypermethylation of the BDNF promoters, can interact with genetic susceptibility, as in the BDNF 66Met carriers (189).

ELS prepares an organism, over the modulation of the HPA axis, for similar adversities during life. This way, a mismatching environment results in an increased susceptibility to psychopathology (131) such as major depression, panic and other co-morbidities. Epigenetics seem to make limbic system structures—mainly the hippocampus and amygdala—more rigid and prone to react depressively and protectively through adulthood. Of clinical significance, a higher occurrence of co-morbidities is usually related to a higher severity of positive and negative symptoms, suicidality, and poorer outcomes (190, 191).

ELS exposure is a negative regulator of BDNF and glucocorticoid receptors (GR) expression in the hippocampus, in the long term, favoring the vulnerability to develop neuropsychiatric disorders, especially upon additional stress exposures (192, 193). An alternative consideration is whether reduced neural capacity leads to a compensatory brain activation that might produce or activate trauma memories. A study of spatial working memory monitored by fMRI found that subjects with schizophrenia had to recruit more cortical regions for the task (194). In this same study, false memory errors were also associated with greater bilateral prefrontal activation. It is plausible that neural strategies to compensate for deficits of perceptual organization, working memory and visuospatial function may lead to a higher recognition of new stimuli as (false) memories. False trauma memory is more frequent among adolescents with posttraumatic stress disorder (PTSD) who experienced childhood sexual abuse (195). Combined, psychosis and childhood sexual abuse may greatly amplify false memories.

It is possible that some of these traumatic experiences are related to PTSD or stress symptoms, as is likely in many cases of abuse. Alternatively, they may be related to direct brain injury, as is likely in many cases of meningitis and encephalitis (196). Most studies examining trauma in schizophrenia risk do not distinguish between events that occur in early childhood versus adolescence, instead identifying events that occur before a determined age (e.g., 16 or 18 years old). However, examination of the timing of trauma suggests that puberty is an important window for distinguishing between anxious and depressive outcomes (197). Future studies examining the timing of traumatic exposures against puberty onset can better elucidate schizophrenia risk in these two populations.

#### Abuse

History of sexual and physical abuse is strongly correlated with greater psychotic symptom severity among adolescents and young adults in clinical high-risk (CHR) cohorts for schizophrenia. Patients from one such cohort reporting sexual abuse as children or adolescents had increased likelihood of transitioning to psychosis (198). Overall, sexual abuse history is more prevalent in these high-risk individuals than the general population (198–204). Physical abuse is also commonly reported by CHR individuals and may be linked with cognitive defects (205–209). Early physical trauma may lead to hyperarousal of the stress response and chronically elevated cortisol levels (210).

Emotional abuse in childhood, including neglect and maltreatment, has negative effects on mental health (211). Perceived discrimination significantly predicts the transition to psychosis, and emotional trauma and bullying are associated with depression, anxiety, and low self-esteem in CHR individuals (205). These various emotional traumas may impair cognitive function by denying a positive, stimulating environment for the developing brain (212, 213).

Physical and emotional trauma in childhood appears to alter stress response. Adults who reported childhood trauma demonstrate blunted cortisol responses, likely an adaptive response to chronic cortisol elevation (211, 214). In schizophrenia, increased stress sensitivity is a potential causal factor (133, reviewed in 215). Mechanistically, chronically increased cortisol may make the hippocampus vulnerable to injury *via* cortisol-induced dendritic restructuring or altered cortisol receptor levels (216–221). Cytokines like IL-6 and TNF-α are elevated in children exposed to trauma and can alter cortisol responses (222, 223). Additionally, the BDNF pathway may be relatively inhibited from chronically elevated cortisol, further promoting hippocampal injury and schizophrenia risk (224).

The gut microbiome is influenced by early childhood trauma and likely influences schizophrenia risk in turn (225). Gastrointestinal distress is frequently associated with early adversity in children, and the gut microbiome appears to influence stress programming in animal models (226–229) (reviewed in 230). Recent studies describe altered microbial patterns in children subjected to adversity, with elevations in *Lachnospiraceae* spp. suggestive of a potential influence on stress sensitivity (231). Additionally, childhood adversity is associated with altered gut microbiota during pregnancy, and may influence observed alterations in inflammatory and GC response to stress, thus contributing to propagation of schizophrenia risk across generations (232). Mechanistically, microglia have an important role in neuroplasticity and neurogenesis and are also sensitive to peripheral inflammation. Gut dysbiosis may negatively influence neurodevelopment through altered microglia activation (228, 233). Future work examining gut microbiome, inflammation, and effects of probiotics in CHR patients may help further elucidate connections between the microbiome, early trauma, and schizophrenia.

#### Infections

Childhood infections are another important risk factor for schizophrenia onset, especially viral CNS infections (234–236) implicating the microbiome. Childhood infections increase schizophrenia risk in a dose-dependent manner and familial liability for infection also increases schizophrenia risk (237). Additionally, hospitalization for severe infection and even outpatient antibiotic treatment in children are related to increased risk for future psychiatric hospitalizations, suggesting a broad impact of childhood infections on mental health (238).

Mechanistically, direct CNS damage from infection or indirect inflammatory damage may drive the increased schizophrenia risk following childhood infections (238). Antibiotic use in response to infection may also drive risk. Several antibiotics including fluoroquinolones are associated with neurotoxicity and psychosis risk (239). In addition to neurotoxic effects, infections and antibiotics can elevate cortisol levels, potentially affecting the stress cascade (240).

The microbiome also likely influences infection risk in schizophrenia. Studies of germ-free mice show that the gut microbiome primes microglia, stimulating viral specific immunity and reducing viral-driven demyelination *via* a TLR4-mediated process (241). Dysbiosis driven by antibiotic use or other factors may therefore increase CNS damage from neuroinvasive viruses and thereby increase schizophrenia risk. Interestingly, one study showed antibiotic treatment during adolescence in mice reduced anxiety-like behavior (99). However, cognitive deficits were shown along with reduced hippocampal BDNF and hypothalamic oxytocin and vasopressin expression so the reduction in anxiety-like behavior is suggestive of negative symptoms.

## Adolescence

Adolescence is the transition from childhood into adulthood that begins with puberty and ends with cessation of physical growth and neural development in the early 20s (242). Puberty broadly impacts mental health, neuroendocrinology, and the microbiome (reviewed in 243). Neurologically, adolescence encompasses improved abstract thinking, reasoning, and knowledge while also seeing a trend toward increased risk-taking behavior. Schizophrenia most frequently develops during adolescence and young adulthood, and the changes that occur during this developmental stage likely participate in shaping schizophrenia risk. As with early childhood, there are concerning signs and exposures during adolescence that are linked to schizophrenia.

### Adolescent Signs

As with early childhood, broad impairments in neuromotor development, cognitive function, and behavior often mark individuals at risk for schizophrenia (reviewed in 154). As the adolescent matures, poor coordination, balance, and perceptual-motor and visual-motor functioning may become more apparent in a subgroup of cases (152, 168, 173). Cognitively, lower intelligence and especially a decrease in intellectual function mark schizophrenia risk (151, 153, 169, 244). There is impairment of individual domains including arithmetic and spelling, formal thought disorders, attention difficulties, increased distractibility, poor executive functioning, and general learning and memory difficulties (152, 153, 169, 173, 245). Behaviorally, aggression, withdrawal, and generally poor social competence and peer relations are also concerning, with psychiatric symptoms including affective flattening and anxiety often present (149, 151, 174, 175, 246, 247).

### Risk Exposures

As discussed earlier, studies of exposures do not usually distinguish between pre-pubescent children and post-pubescent adolescents. The aforementioned exposures of sexual, physical, and emotional abuse as well as infection similarly convey schizophrenia risk among adolescents. However, trauma may have different long-term outcomes post-puberty, and its potential effect on schizophrenia risk merits further study. Additionally, the increased risk-taking behavior exhibited at this stage may be influenced by early trauma and influence further trauma exposures. New exposures, such as recreational drug use, may also contribute to schizophrenia risk.

### Recreational Drug Use

Recreational drugs exploration is frequent in adolescence and many carry a significant risk for psychosis, particularly cannabis. By their first psychotic episode, approximately half of patients will have a history of cannabis use and one-third meet criteria for cannabis use disorder (248). Alcohol use is similarly high among individuals who have experienced their first psychotic episode, and there is elevated use of cocaine, amphetamine, barbiturate, and other drugs. Cause and effect associations of cannabis and psychosis are well described, although some schizophrenia-susceptible individuals may self-medicate to reduce the anxiety surrounding the presentation of schizophrenia symptoms, with this drug-seeking behavior may further exacerbate their risk for the disorder (249). Chronic exposure to tetrahydrocannabinol (THC), an active ingredient in cannabis, can disrupt neurodevelopmental maturation dependent on endocannabinoid pathways and may lead to overactivation of a pro-hallucinogenic pathway of 5-HT2A receptors, which may promote schizophrenia onset in susceptible individuals (250).

Substance abuse can dysregulate the HPA axis. Alcohol and nicotine use induce cortisol production, and long-term use can cause chronic cortisol elevation and dysregulation similarly to previously described trauma (251–254). Additionally, the gut microbiome is dysregulated by psychostimulants, alcohol, and opioids (255–259) (reviewed in 260). Microbiome influences on addiction are an active area of research. Microglial function is shaped by the microbiome and altered by drugs of abuse (233, 261). Likewise, BDNF dysregulation by dysbiosis is associated with altered behavioral response to cocaine and alcohol (256, 262, 263). While more work is needed to establish causal relationships, these findings suggest multiple ways in which the microbiome may influence addiction behaviors.

## Young Adulthood

The transition from adolescence to adulthood occurs during the 20s (242). This transition is typically marked by completion of education and transition to complete independence, which can increase stress in a young adult’s life. Onset of schizophrenia typically occurs around this life transition, peaking at 18 to 25 years old in men and 25 to 35 years old in women, with 80% of cases initially presenting before 40 years of age (264–266). The age of schizophrenia onset may be related to immune activation and stress. Interestingly, inflammatory diseases including inflammatory bowel disease, multiple sclerosis, and some autoimmune diseases tend to initially present in young adulthood (264–266). Gut dysbiosis and cortisol dysregulation are observed in many autoimmune diseases and disruptions to these systems in early adulthood likely influence schizophrenia onset as well (reviewed in 267, 268). First-episode schizophrenia patients have well-documented inflammatory disturbances, such as cytokine elevations and microglial activation (reviewed in 269).

Metabolic disturbances, including glucose intolerance, insulin resistance, and hyperglycemia, also frequently present in this age group and are more common among antipsychotic and naïve first-episode schizophrenic patients compared to the general population (270, 271). These changes may promote schizophrenia onset through persistent inflammatory effects. Stress-related cortisol elevations and gut dysbiosis both contribute to metabolic disturbances, suggesting alternative pathways that influence schizophrenia risk (272, 273). The microbiota also regulate adult neuroplasticity and microglia activation (233, 274).

### Aerobic Exercise: A Potentially Protective Factor

While a number of risk factors for schizophrenia are identified, recent evidence points to protective factors. Specifically, aerobic exercise (AE) is hypothesized to play an important protective role against stress induced effects. AE induces a cascade of molecular and cellular processes that support brain plasticity and growth of new vasculature and trigger the processes through which neurotrophins mediate neural plasticity (reviewed in 275–278). Among neurotrophins, BDNF is the most susceptible to regulation by physical exercise (279–281), with synthesis and release into the blood circulation increasing in a dose-response manner (282, 283). Consistent with these findings, Voss et al. (284, 285) found increased connectivity between the bilateral parahippocampus and the bilateral middle temporal gyrus was linked to BDNF increase in AE subjects. A recent meta-analysis (286) of 29 studies (N = 1111 healthy subjects) examined the effect of exercise on BDNF in three exercise paradigms: 1) a single session of exercise; 2) a session of exercise following a program of regular exercise; and 3) resting BDNF levels following a program of regular exercise. Results demonstrated a moderate effect size for increases in BDNF following a single session of exercise (Hedges’ g = .46, p < .001). Further, regular exercise intensified the effect of a session of exercise on BDNF levels (Hedges’ g = .59, p = .02). Finally, results indicated a small effect of regular exercise on resting BDNF levels (Hedges’ g = .27, p = .005). Examination of moderator effects across paradigms found that subjects’ age was not significantly related to changes in BDNF following exercise, but sex significantly moderated the effect of exercise on BDNF levels, such that studies with more women showed less BDNF change resulting from exercise.

Consistent with these reports, findings indicate individuals with schizophrenia tend to have highly sedentary lifestyle characterized by low aerobic fitness which was highly correlated with poor cognitive functioning and symptoms (287). These findings parallel reports among individuals at clinical high risk for psychosis indicating lower levels of fitness, less physical activity, as well as more barriers to exercise (288–292). Yet, a pilot AE RCT indicated engagement in AE led to 11.0% increase aerobic fitness (293) as well as BDNF vs. a 1.9% in the TAU subjects (294) (reviewed in 295). A hierarchical multiple regression analysis indicated that, after controlling for age, sex, changes in anti-psychotic and SSRIs, and changes in menstrual cycle phase, BDNF changes independently predicted changes in cognitive function (b = .38, t = 2.06, p = .05) (296). Notably, improvements in cognitive functioning were associated with intensity of AE activity (294).

Exercise alters the composition and functional capacity of the gut microbiome independent of diet (reviewed by 28). As the effects of AE on BDNF production are further studied in schizophrenia, examination of how the microbiome influences this pathway may be illuminating.

## Potential Mechanisms

Although some stress exposure is essential for growth and development, stress that overwhelms adaptive capacities has adverse physiological consequences, as initially described in 1938 by Hans Selye (297). The initial stress axis model included direct and feedback interactions among the hypothalamus (release of corticotropin-releasing factor), pituitary (ACTH), and adrenal glands (cortisol), which was then expanded by Sapolsky’s “glucocorticoid cascade hypothesis” (298) to encompass catecholamines and other interacting mediators of adaptation in addition to GCs. This model must now be widened to include the central influence of the microbiota on the initial programming of the stress axis and ongoing bidirectional effects that influence stress responding. The communication pathways between the gut and brain includes the vagal nerve, through which some microbial species invoke anxiolytic effects of some species (299). Enteroendocrine cells secrete biologically active peptides, including galanin, which stimulates the central HPA axis leading to increased adrenal cortisol secretion, and ghrelin which has similar effects linked to nutritional and metabolic conditions (300, 301) (reviewed in 302, 303). Reciprocally, even short durations of stress impact the relative proportions of phyla in the microbiota mediated through neuroendocrine and autonomic nervous system activity (304). The neuro-immuno-endocrine pathways linking the gut and brain include afferent and efferent neural pathways, immune effects, bi-directional neuroendocrine signaling and by alterations in intestinal permeability, critically influenced by relative proportions of microbiota species, as shown in **Figure 1**.

Examined as a whole, broad pathways through which the gut may influence stress and schizophrenia risk include cytokine-driven global inflammatory modifications, stress hormone metabolism, microglial activation, neuroplastic regulation, direct infection, and other nervous system activity as described above. Given schizophrenia risks at key developmental stages also coincide with microbiome development and associated changes, examining these pathways across development may be especially poignant. During the perinatal period, as the brain and HPA axis develop, dysbiosis in mother and child is influenced by multiple factors including infection and stress and in turn may influence the brain and HPA axis. As the child continues to grow and develop, the microbiome continues to adapt and change. While stressors including psychic and physical trauma, recreational substance use, inflammatory diseases, metabolic disturbances, and AE have been previously understood in context of neuroendocrine pathways, these events also affect the microbiome which in turn likely feed back into stress and neurodevelopment pathways. When viewed as one interconnected system, the ways microbial, endocrine, and neurological pathways influence each other across development should improve our understanding of schizophrenia risk and perhaps offer novel treatment methods. While current knowledge rests largely on germ free, antibiotic treated or probiotic supplemented animal models, the field is finally advancing to human studies.

## Conclusion

Our understanding of schizophrenia risk has evolved over the past century as technological improvements have made better research methods possible. Recent decades demonstrate the profound impact that neuroendocrine pathways have on schizophrenia risk across human development. The microbiome represents one of the newest frontiers in research that is broadly impacting healthcare. Recent work has already demonstrated many interactions between schizophrenia risk, neuroendocrinology, and the microbiome, but there are unexplored areas throughout development where further interactions likely occur. Thus, future work examining schizophrenia risk must continue to incorporate the crosstalk between the neuroendocrine pathways and the microbiome.

## Author Contributions

KH, JL, CC, DK, TK, and DM all contributed to writing and editing manuscript.

## Funding

This work is supported by NIMH R01 MH110623 and P50MH115843 (DK); NIMH R01 MH107558 and R01 MH115332 (CC); NIMH R01 MH110418 (DM).

## Conflict of Interest

The authors declare that the research was conducted in the absence of any commercial or financial relationships that could be construed as a potential conflict of interest.
